# DFT Study on the Mechanism of Iron-Catalyzed Diazocarbonylation

**DOI:** 10.3390/molecules25245860

**Published:** 2020-12-11

**Authors:** Tímea R. Kégl, László Kollár, Tamás Kégl

**Affiliations:** 1 Department of Inorganic Chemistry and MTA-PTE Research Group for Selective Chemical Syntheses, University of Pécs, H-7624 Pécs, Hungary; trkegl@gamma.ttk.pte.hu (T.R.K.); kollar@gamma.ttk.pte.hu (L.K.); 2 Department of Inorganic Chemistry and János Szentágothai Research Centre, University of Pécs, H-7624 Pécs, Hungary

**Keywords:** iron-carbonyls, diazocarbonylation, DFT

## Abstract

The mechanism of the carbonylation of diazomethane in the presence of iron–carbonyl–phosphine catalysts has been investigated by means of DFT calculations at the M06/def-TZVP//B97D3/def2-TZVP level of theory, in combination with the SMD solvation method. The reaction rate is determined by the formation of the coordinatively unsaturated doublet-state Fe(CO)_3_(P) precursor followed by the diazoalkane coordination and the N_2_ extrusion. The free energy of activation is predicted to be 18.5 and 28.2 kcal/mol for the PF_3_ and PPh_3_ containing systems, respectively. Thus, in the presence of less basic P-donor ligands with stronger π-acceptor properties, a significant increase in the reaction rate can be expected. According to energy decomposition analysis combined with natural orbitals of chemical valence (EDA–NOCV) calculations, diazomethane in the Fe(CO)_3_(phosphine)(*η*^1^-CH_2_N_2_) adduct reveals a π-donor–π-acceptor type of coordination.

## 1. Introduction

Ketenes are important and versatile intermediates in synthetic organic chemistry [1,2,3,4,5]. They can form various carboxylic acid derivatives, such as esters, anhydrides, and amides reacting readily with alcohols, carboxylic acids, or primary amines, respectively [6]. A notable category of ketene reactions is their facile [2+2] cycloaddition with alkenes, dienes, or imines [7,8]. The reactive ethoxycarbonyl ketene can be trapped in situ by various scavengers such as alcohols, amines, and imines affording the corresponding malonic acid derivatives or β-lactams, respectively [9]. Moreover, in the co-catalyzed domino reaction of ethyl diazoacetate (EDA) with CO, in the presence of ferrocenylimines, the synthesis of unsaturated malonic acid derivatives has also been reported [10,11].

**Scheme 1:**
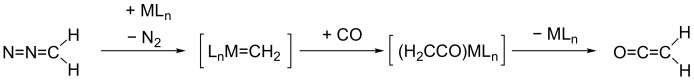
Catalytic transformation of diazomethane in the presence of transition metal complexes and carbon monoxide.

One straightforward way leading to ketenes is the metal-mediated substitution of the diazo group in diazoalkanes, as depicted in Scheme 1. The first metal-mediated example for the carbonylation of diazoalkanes was reported by Rüchard and Schrauzer in 1960 [12]. Nickel tetracarbonyl served as both catalyst precursor and CO surrogate for converting diazomethane, diphenyldiazomethane, and ethyl diazoacetate to the corresponding ketenes. In the following decades, no other similar results were published, despite the potential synthetic importance of the diazocarbonylation reaction.

A crucial step in the reaction is the formation of the ketene complex from the carbenoid that is afforded after the N_2_ extrusion. Grotjahn and co-workers studied the interconversion of diphosphine-substituted iridium carbonyl–carbene complexes by intramolecular C=C bond cleavage/formation [13,14]. The equilibrium of the reversible step was predicted to be on the side of the carbonyl–carbene system, whereas for the analogous rhodium-containing systems the equilibrium was found to be on the ketene side.

The carbonylation of ethyl diazoacetate and trimethylsilyl diazomethane was thoroughly investigated in the presence of cobalt catalysts in [15,16]. For Co_2_(CO)_8_, as a catalyst precursor, the reaction leading to diethyl malonate was found to occur in two different cycles. In the first one, the coordinatively unsaturated Co_2_(CO)_7_ serves as the active catalyst, which forms the bridging carbonyl–carbene complex Co_2_(CO)_7_(CHCOOEt) after its reaction with EDA [17,18]. The catalytic cycle can go on with an intramolecular ketene formation and eventually the release of ketene, or with the dissociation of a terminal CO ligand giving rise to the unsaturated Co_2_(CO)_6_(CHCOOEt), which is also able to react with diazoalkanes, opening the second catalytic cycle [19]. In this pathway, a stable Co_2_(CO)_6_(CHCOOEt)_2_ dicarbene intermediate is formed, which can also couple one terminal carbonyl ligand affording complexes with ethoxycarbonylketene ligands. After dissociation, the ketene reacts with the trapping agent ethanol to form diethyl malonate as the final product [20].

Triphenylphosphine-substituted cobalt carbonyl complexes were found to be even more active precatalysts in the carbonylation of EDA. With two equivalents of PPh_3_, the presence of the ion pair [Co(CO)_3_(PPh_3_)_2_][Co(CO)_4_] could be observed, which was assumed to interconvert to the corresponding radical pair under catalytic conditions [21].

The palladium-catalyzed diazocarbonylation, the subsequent domino reaction leading to β-lactams, and their mechanisms were investigated by Wang and co-workers [22]. For the computational studies, Pd-CO-ethylene complexes were postulated as model catalysts. It was suggested that the isomerization of the C=N bond of the initially formed zwitterionic intermediate took place, which would give an explanation for the predominant formation of *trans*-β-lactams.

The mechanism of diazo activation and carbonylation was investigated in the presence of homoleptic and phosphine substituted nickel carbonyl catalysts in [23]. Substitution of CO by PH_3_ resulted in a decrease of the activation barrier, which was attributed to the combined catalyst formation/N_2_ extrusion step. In contrast to the cobalt-containing systems, the mostly exergonic step was the carbene–carbonyl coupling instead of the formation of the carbenoid.

The goal of this work is to unravel the mechanism of diazomethane carbonylation in the presence of iron–carbonyl–phosphine complexes. The secondary purpose of this paper is to interpret the electronic structure of reaction intermediates and classify their donor–acceptor character using energy decomposition analysis combined with the natural orbitals of chemical valence (EDA–NOCV) as well as the natural bond orbital (NBO) methodology.

## 2. Results and Discussion

One of the straightforward synthetic routes leading to ketene is the carbonylation of diazoalkanes; that is, the replacement of the N_2_ moiety by carbon monoxide. The resulting carbenoid then transfers to the corresponding ketene complex via intramolecular carbene–carbonyl coupling. In all cases, the first step of the metal-mediated reaction is the coordination of diazoalkane to the metal.

### 2.1. Reaction of Diazomethane with Phosphine Substituted Iron Carbonyls

**Figure 1:**
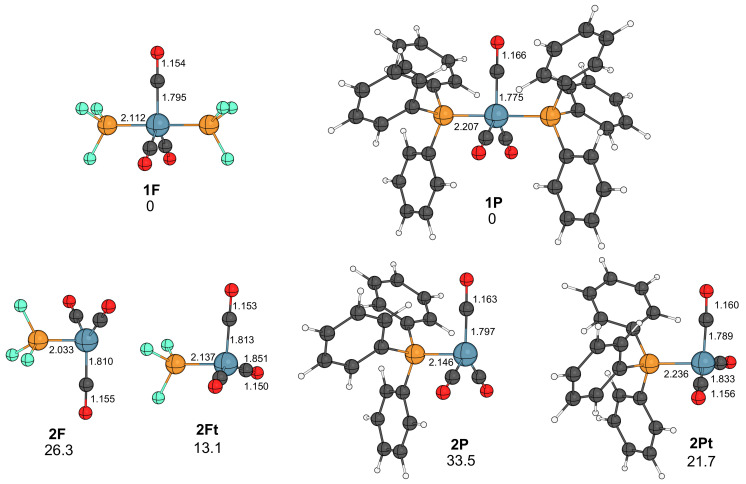
Computed structures of coordinatively saturated Fe(CO)_3_(P)_2_ and unsaturated Fe(CO)_3_(P) types of complexes. Gibbs free energy values are given in kcal/mol and bond lengths are in Å. The free energy values are relative to **1F** (for the phosphorus-trifluoride-containing complexes) and **1P** (for the triphenylphosphine-containing complexes).

The precursor for the catalytic reaction is the trigonal bipyramidal *trans*-[Fe(CO)_3_(L)_2_] complex, which releases one of its ligands, thereby enabling the coordination of diazoalkane on the vacant site. Complex **1F** has a D_3*h*_ symmetry, which is reduced to C_3_ in **1P** because of the lack of symmetry planes. As expected, the strong acceptor property of the PF_3_ ligand results in a shorter Fe−P, C=O, and an elongated Fe−C bond in **1F**, as compared to those in the complex **1P** with PPh_3_. Since the dissociation of the carbonyl ligands is significantly more endergonic than that of the phosphines, only the formation of the unsaturated Fe(CO)_3_(L) complexes was examined. By the removal of one phosphine ligand, the resulting coordinatively unsaturated complexes **2F** and **2P** keep the symmetry of their “parent” complexes. In addition to these singlet state species, triplet structures were found, and they proved to be more stable in terms of free energy than their singlet state counterparts. The free energy difference is 13.2 kcal/mol between **2Ft** and **2F**, while **2Pt** is more stable than **2P** by 11.8 kcal/mol. The stability difference for these Fe(CO)_3_(L) types of complexes shows some similarity with that obtained for the unsaturated iron tetracarbonyl, where ^3^[Fe(CO)_4_] proved to be more stable by ca. 8 kcal/mol than the singlet state tetracarbonyl, according to Harvey and Aschi [24]. In the triplet structures, the Fe−P distances are slightly more elongated than those in the singlet structures. Instead of a threefold axis, only one symmetry plane remains in **2Ft** (C_s_ symmetry), as one of the OC−Fe−CO angles is increased to 148.7°. The symmetry is C_1_ for **2Pt**, with a OC−Fe−CO angle of 148.1°. The structures of the initial phosphine substituted carbonyl complexes are depicted in Figure 1.

**Figure 2:**
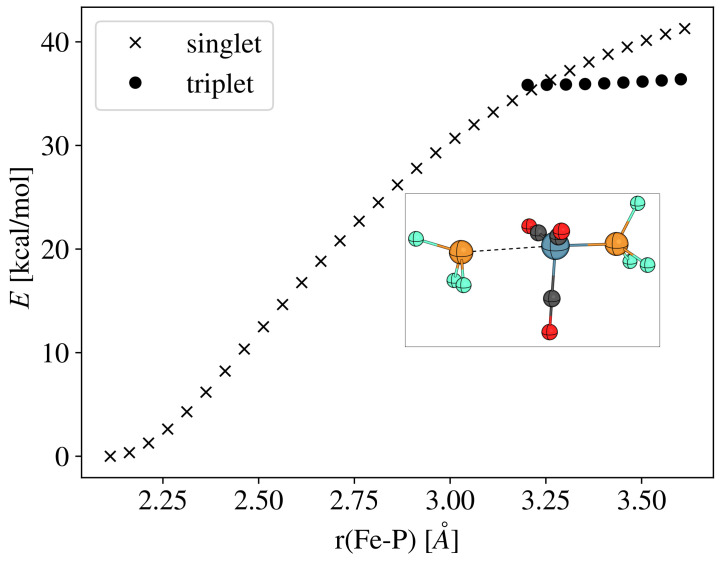
Energies of the partially optimized singlet and triplet Fe(CO)_3_(PF_3_)_2_ relative to the overall singlet minimum, at various Fe–P distances. The computed structure in the inlet represents the minimum energy crossing point (MECP).

Thus, in both cases, the triplet structures are preferred for the unsaturated Fe(CO)_3_(L) complexes, meaning that the dissociation of one phosphine ligand is expected to take place via a spin change. In Figure 2, the nearest approximation to the adiabatic transition state (that is, the minimum energy crossing point) is depicted for the phosphorus-trifluoride-containing system. On the triplet potential energy surface, we found no real stable diphosphine tricarbonyl complex, as in the local minimum of the saturated species, the iron–phosphorus bond distance exceeds 3 Å (see Figure S1 in the Supplementary Material). The spin state change takes place at a Fe–P distance of 3.23 Å. Another spin state change is expected to take place during the coordination of diazomethane.

To check the dependence of the spin state upon the functional, the free energy difference between the triplet and singlet states of complexes Fe(CO)_3_(P) has been computed as well, employing the B3LYP and the TPSS functionals. It is known that in some cases nonhybrid functionals prefer the low-spin state, whereas B3LYP overstabilizes the triplet state [25]. Here, the free energy difference between **2F** and **2Ft** was 6.5 and 14.9 kcal/mol for TPSS and B3LYP, respectively, in favor of the triplet structure. For the analogous complexes with triphenylphosphine, **2Pt** was more stable than **2P** by 5.2 and 19.0 kcal/mol for TPSS and B3LYP, respectively, in terms of free energy. These results support the observation found at the M06//B97D3 level (that is, M06 energies on B97D3 geometries) that the catalytically active species is the triplet Fe(CO)_3_(P) for both P-donor ligands.

**Scheme 2:**
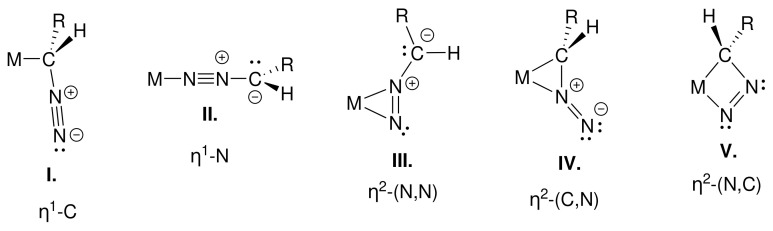
Most common coordination modes for diazoalkanes bound to a single transition metal center.

**Scheme 3:**
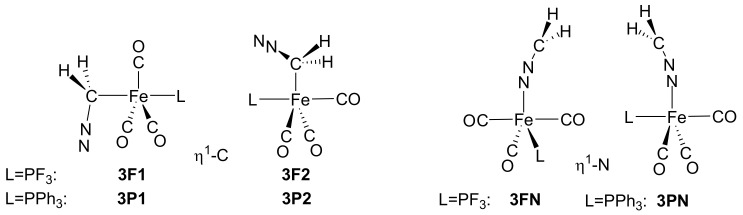
Schematic representation of Fe(CO)_3_(L)(diazomethane) complexes with $\eta^{1}$-C and $\eta^{1}$-N coordination.

For metal–diazoalkane complexes, various coordination types are known in the literature. The most common ones are depicted in Scheme 2. The possibilities I. to V. were thoroughly checked for both phosphine ligand types. No $\eta^{2}$ coordination to the Fe(CO)_3_(PR_3_) moiety was found; that is, the diazo coordination is predicted to proceed via $\eta^{1}$ diazo complexes. Scheme 3 illustrates the $\eta^{1}$-N diazo species, as well as the $\eta^{1}$-C complexes, where diazomethane can take either the axial or the equatorial position.

**Figure 3:**
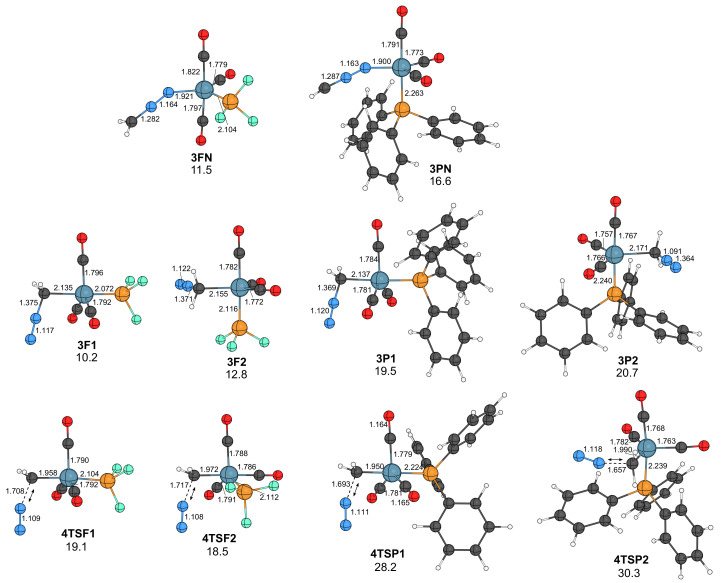
Computed minimum structures associated with the diazo coordination and activation steps, as well as transition states for the dinitrogen extrusion step. Bond distances are in Å and Gibbs free energy values are given in kcal/mol. Free energies are relative to **1F** and **1P**.

Probably because of steric reasons, only one example each was found for the $\eta^{1}$-N diazo adducts; the PF_3_ ligand is equatorial in **3FN**, whereas PPh_3_ occupies the axial position in **3PN**. For the $\eta^{1}$-C species, diazomethane can adopt both axial (**3F1** and **3P1**) and equatorial (**3F2** and **3P2**) positions; the axial complexes (with the P-donor ligand in *trans* position) are more stable for both PF_3_ and PPh_3_ with a free energy difference of 2.6 and 1.2 kcal/mol, respectively. Complex **3FN** is less stable than **3F1** by 1.3 kcal/mol, however, **3PN** is the most stable adduct in the presence of triphenylphosphine as it is more stable by 2.9 kcal/mol in comparison to **3P1**. The computed structures of the diazo adducts are depicted in Figure 3.

**Figure 4:**
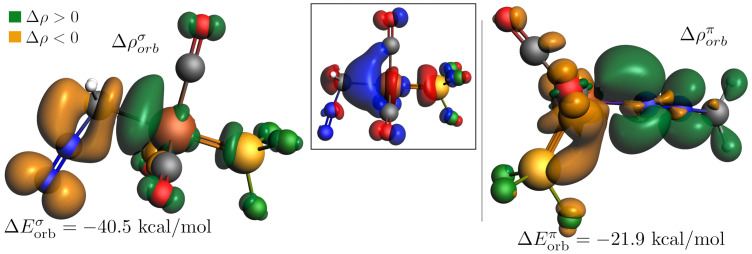
Natural orbitals of chemical valence (NOCV) deformation densities associated with the dominant interaction of the diazomethane complexes **3F1** (left) and **3FN** (right). The inlet shows the acceptor NOCV orbital of **3F1** with the eigenvalue of 0.698.

The dominant interaction between the metal and diazomethane is remarkably different for the two $\eta^{1}$ coordination types of diazomethane, as it is unraveled by energy decomposition analyses within the framework of the natural orbitals of chemical valence method (EDA–NOCV) [26,27,28,29] and depicted in Figure 4. For the $\eta^{1}$-N case, the leading interaction is the back-donation to the diazo moiety with involvement of *d* orbitals of iron extended with the lone pair of phosphorus. The main orbital interaction of complex **3F1**, however, stems from the $\pi_{\mathrm{CN}}$ orbital; that is, even though the coordination type is $\eta^{1}$, the interaction is π-donor in character, whereas the acceptor NOCV is a combination of an empty sd hybrid on Fe, and the mixture of $\sigma_{\mathrm{PF}}^{*}$ and $\pi_{\mathrm{CO}}^{*}$ orbitals. It is interesting to note that the leading orbital interaction in **3F1** is twice as strong (−40.5 kcal/mol) as that in **3FN** (−21.9 kcal/mol).

The adducts with diazoalkane undergo N_2_ extrusion resulting in metal carbenoids. The transition states (**4TSF1**, **4TSP1**, **4TSF2**, and **4TSP2**) describing this process are originated from the respective $\eta^{1}$-C complexes. For the PF_3_-containing species, the equatorial pathway is slightly preferred (by 0.6 kcal/mol in terms of free energy), even though the corresponding diazo adduct is less stable than the axial one. On the other hand, the activation free energy difference is larger in the presence of PPh_3_, and the axial pathway is preferred by 2.1 kcal/mol. For the $\eta^{1}$-N diazo complexes, it is also possible, in principle, to cleave the C–N bond with the attack of an unsaturated complex on the diazo methylene group, as was described by Milstein and co-workers for Rh–diazo species [30]. A similar pathway was reported for the ketene formation from ·Cr(Cp)(CO)_2_(NNCH_2_) with the addition to the ·Cr(Cp)(CO)_3_ radical [31]. This pathway was checked only for PF_3_ with a transition state describing the reaction of **3FN** with **2Ft** (see Figure S2 in the Supplementary Material), and the activation free energy was found to be 20.3 kcal/mol; that is, this route is predicted to be the least favored compared to those involving the $\eta^{1}$-C complexes. On the triplet potential energy hypersurface (PES), the addition of diazomethane to the unsaturated phosphine–carbonyl complexes was examined as well. The relative free energies of the adducts exceeded those of the singlet state transition states by 5.9 and 3.2 kcal/mol for PF_3_ and PPh_3_, respectively, therefore we did not follow the triplet pathways of the N_2_ extrusion.

### 2.2. Formation of Ketene Complexes

**Figure 5:**
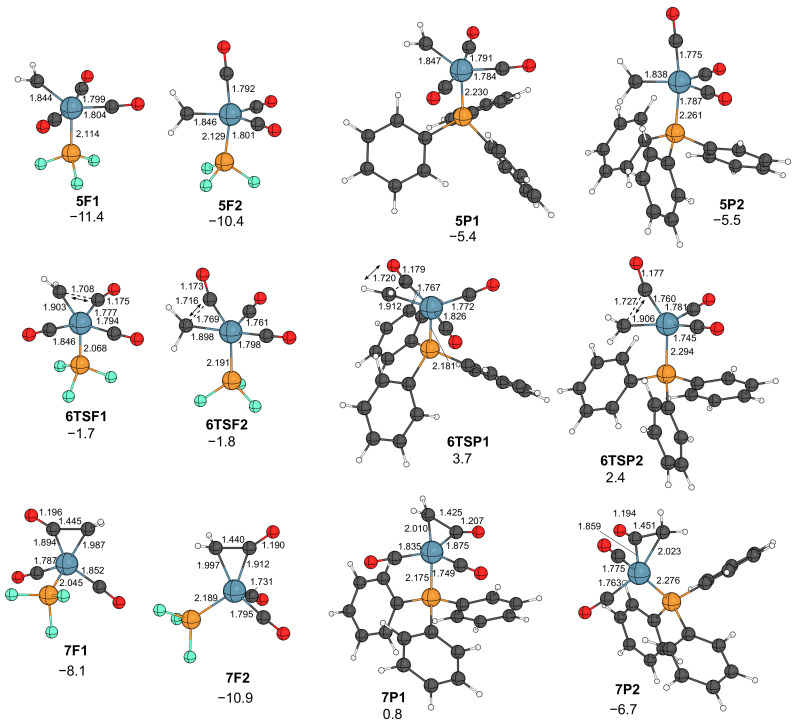
Computed minimum structures and the transition states describing the carbene–carbonyl coupling for the PF_3_ and PPh_3_ pathways for the CO uptake step leading to coordinatively saturated ketene complexes. Bond distances are in Å, Gibbs free energy values are given in kcal/mol, and they are relative to **1F** and **1P**.

The N_2_ extrusion is exergonic in all cases, resulting in the carbenoids **5F1** and **5P1** for the axial pathway and **5F2** and **5P2** for the equatorial pathway via transition states **4TSF1**, **4TSP1**, **4TSF2**, and **4TSP2**, respectively (Figure 3). Complex **5F1** is somewhat more stable thermodynamically than **5F2**, however, there is no significant difference in stability for the triphenylphosphine-containing species. From the interaction of the methylene group with an adjacent CO, coordinatively unsaturated ketene complexes are formed. The coupling follows the route through the **6TSF1** and **6TSP1** transition states for the axial, and **6TSF2** and **6TSP2** transition states for the equatorial reaction channels. On the axial pathway, complexes **7F1** and **7P1** have the P-donor ligand off the plane spanned by the ketene and carbonyl ligands. Ligands PF_3_ and PPh_3_, on the other hand, are in-plane in complexes **7F2** and **7P2**. Species **7F1** and **7F2** are close to each other in terms of free energy, whereas **7P1** is notably less stable than **7P2** (Figure 5).

**Figure 6:**
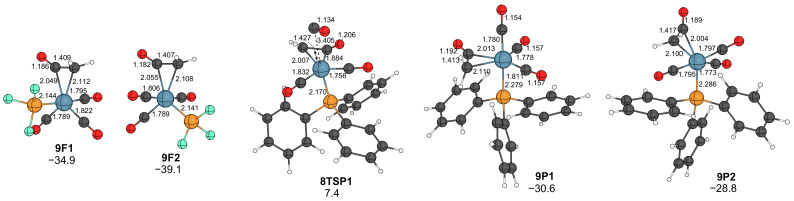
Computed minimum structures of the coordinatively saturated ketene complexes and transition states associated with the CO uptake in the presence of triphenylphosphine. Bond distances are in Å, Gibbs free energy values are given in kcal/mol, and they are relative to **1F** and **1P**.

The coordinatively unsaturated ketene complexes are prone to taking up one CO from the external carbon monoxide atmosphere. For the phosphorus-trifluoride-containing complexes, the CO coordination is barrierless, similar to the ketene complex with PPh_3_ on the equatorial pathway (**7P2**). For the axial pathway, however, the CO coordination proceeds via a transition state (**8TSP1**) with a free energy barrier of 6.6 kcal/mol. The formation of the saturated complexes **9F1**, **9F2**, **9P1**, and **9P2** is highly exergonic in all cases. In the presence of PF_3_, species **9F2** is more stable, where the P-donor ligand is in the Fe–ketene plane (Figure 6).

**Figure 7:**
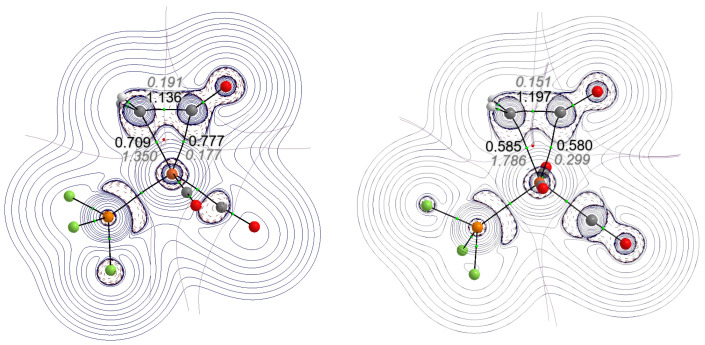
Contour-line diagrams of the Laplacian distribution ($\nabla^{2}\rho$(**r**)) of the unsaturated (**7F2**, left) and saturated (**9F2**, right) ketene complexes with PF_3_ ligand.

By inspection, there is no apparent difference in the charge distribution around the bound ketene in **7F2** and **9F2** (Figure 7). In accordance, however, with the larger Fe–C distances in **9F2**, the delocalization indices for the iron–carbon interactions are smaller, as compared to those in the unsaturated **7F2**. Consequently, the C–C delocalization index is larger in **9F2**, indicating a stronger carbon–carbon interaction. On the other hand, the C–C bond ellipticity is smaller in the saturated complex, which implies a somewhat less pronounced double-bond character. It is interesting to note that in both cases the iron–carbon bond ellipticity is fairly high for the Fe– –C_carbonyl_ interaction (higher than that for the C– –C bond) and extremely high (1.350 for the unsaturated and 1.786 for the saturated complex) for the interaction between the iron center and the terminal carbon.

**Figure 8:**
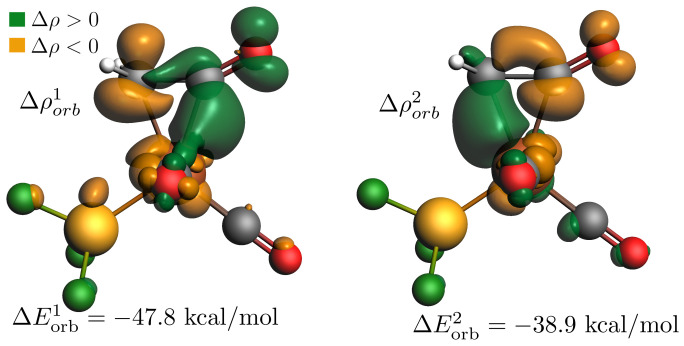
NOCV deformation densities associated with the dominant two interactions of the ketene complex **9F2**.

Visualizing the charge flow within the saturated ketene complex **9F2**, the most appealing feature to note is that the prevailing charge transfers are not separated between the metal-containing and the ketene fragments, but a significant part takes place within the ketene fragment (Figure 8). The main deformation density component ($\Delta \rho_{orb}^{1}$) is associated with an orbital interaction energy component of −47.8 kcal/mol, whereas the second-largest orbital interaction energy component ($\Delta \rho_{orb}^{2}$) is −38.9 kcal/mol. These two components are almost complementary in terms of shape: as in $\Delta \rho_{orb}^{1}$ the origin of the charge concentration is the lone pair of π symmetry on the terminal carbon, while in $\Delta \rho_{orb}^{2}$ the charge flow starts from the carbonyl group of the ketene fragment.

**Figure 9:**
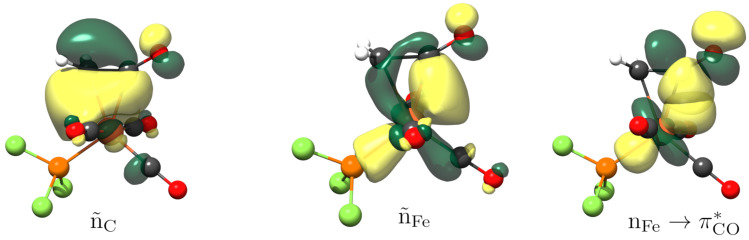
Natural localized molecular orbitals (NLMOs) for the leading donating interaction (left), and for the leading back-donating interaction (middle). On the right side, the dominant interaction of the NBOs for the back-donation is depicted.

To gain further insight into the coordination properties of ketene bound to the Fe(CO)_3_(CF_3_) fragment, NBO calculations were performed with the goal of finding the leading π-donor and π-acceptor interactions. Natural localized molecular orbitals (NLMOs) are based on parent NBOs extended with delocalization tails, thereby representing electron pairs with an occupation number of 2 [32]. The parent NBO of the NLMO representing the π-donor pair is the lone pair of the terminal carbon, mainly based on the $p_{z}$ natural atomic orbital (NAO). To a smaller extent, the $\pi^{*}$ orbitals of the perpendicular CO ligands are involved in the π-donor interaction. The main source of the back donation interaction is the lone pair of iron, which is an out-of-phase hybrid of the 3$d_{x^{2}-y^{2}}$ and the 3$d_{z^{2}}$ NAOs, interacting with the $\pi_{CO}^{*}$ NBO (Figure 9).

### 2.3. Overall Reaction Mechanism

**Figure 10:**
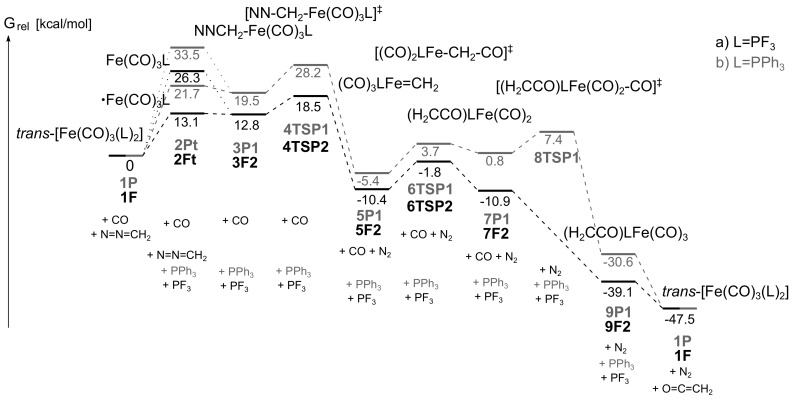
Free energy profile of the diazocarbonylation reaction catalyzed by iron–carbonyl–phosphine complexes. Free energy values are given in kcal/mol.

The free energy diagram of the more preferred pathways for the triphenylphosphine and phosphorus trifluoride complexes are shown in Figure 10. The coordination strength of ketene in the saturated complexes **9F1**, **9F2**, **9P1**, and **9P2** are still not enough to establish stable ketene complexes, which could be resting states on the respective potential energy hypersurfaces. Thus, the dissociation of ketene and the coordination of CO leads to initial complexes **1F** and **1P** with free energy changes of −8.4 and −16.9 kcal/mol, respectively, closing the catalytic cycle.

The reaction mechanism itself shows no substantial difference as compared to those for cobalt- and nickel-containing systems; the formation of the coordinatively unsaturated active catalyst is followed by the diazo coordination and N_2_ extrusion, providing the combined rate-limiting step throughout the reaction. The carbene–carbonyl coupling is reasonably fast, resulting in the coordinatively unsaturated ketene complexes. The CO uptake is strongly exergonic in all cases, resulting in the saturated ketene complexes, and the dissociation of the ketene ligand is also exergonic.

## 3. Computational Details

All the structures were optimized without symmetry constraints with tight convergence criteria using the programs ORCA 4.2.1 [33], with the exchange and correlation functionals developed by Grimme [34] containing the D3 empirical dispersion correction with Becke and Johnson damping [35], and denoted as B97D3. For all the atoms the def2-TZVP basis set [36] was employed.

On the equilibrium geometries, the energies have been recomputed with the M06 functional employing the SMD solvation method with dichloromethane as the solvent ($\varepsilon_{0}$ = 8.93). The thermal corrections to the Gibbs free energy were obtained at the B97D3 level.

Natural bond orbital (NBO) analyses have been performed by the GENNBO 7.0 program [37]. Quantum theory of atoms in molecules (QTAIM) analyses of the wave function [38] were carried out with the AIMAll software [39]. For both methods, the input files were created with the Gaussian 16, Revision C.01 [40] package. For the EDA–NOCV calculations, the ADF 2019 software was used [41] employing the PBEPBE functional in combination with the triple-ζ STO basis set for all atoms with one set of polarization functions (denoted as TZP) and a small frozen core.

## 4. Conclusions

The mechanism of iron-catalyzed diazocarbonylation has been investigated by means of density functional calculations at the M06/def-TZVP//B97D3/def2-TZVP level of theory in combination with the SMD solvation method. The results disclosed herein can be summarized as follows.

- In the presence of phosphine, the course of the reaction resembles that obtained for the nickel–carbonyl catalysts; that is, the $\eta^{1}$–diazoalkane complexes lose N_2_, and the resulting carbenoids undergo carbene–carbonyl coupling affording coordinatively unsaturated ketene complexes.
- The active catalysts for the iron-catalyzed diazocarbonylation are predicted to be the triplet state Fe(CO)_3_(P) complexes, which are formed via a spin change from the corresponding singlet state Fe(CO)_3_(P)_2_ species. The coordination of the diazoalkane proceeds through another spin change.
- As for the other metals, the rate-limiting step is the combination of catalyst formation, diazo coordination and the exergonic N_2_ extrusion. The carbene–carbonyl coupling is slightly exergonic for PF_3_ and endergonic for PPh_3_. The CO uptake, leading to the coordinatively saturated ketene complexes, is exergonic, as well as the dissociation of the ketene ligand from the iron center.
- Electron-withdrawing P-donor ligands, such as phosphorus trifluoride, are predicted to increase the reaction rate, in comparison to that obtained with triphenylphosphine.
- Diazomethane can follow $\eta^{1}$-C or $\eta^{1}$-N coordination; both types of adducts are close to each other in terms of relative free energy.
- According to EDA–NOCV calculations, the charge flow cannot be separated clearly between the ketene and the metal-containing fragment. The more localized NBO approach shows, however, that the main source of the π-donor interaction is the lone pair of the terminal carbon, mainly based on the 2$p_{z}$ natural atomic orbital, whereas the back-donation is based mainly on the lone pair of iron, which is an out-of-phase hybrid of the 3$d_{x^{2}-y^{2}}$ and the 3$d_{z^{2}}$ natural atomic orbitals, interacting with the $\pi_{CO}^{*}$ orbital of bound ketene.

Thus, according to the calculations, the phosphine-modified iron carbonyl systems are expected to work as catalysts in diazocarbonylation, and possibly in domino reactions based on the catalytic preparation of ketenes. Work is underway in our laboratory to optimize the reaction conditions and to find the most appropriate P-donor ligands for the catalytic transformations.

## Supplementary Materials

The following are available online. Figure S1: Computed structure of the triplet state adduct Fe(CO)_3_(PF_3_)_2_; Figure S2: Computed structure of the triplet transition state for the associative pathway between complex **3F1** and ·Fe(CO)_3_(PF_3_) leading to Fe(CO)_3_(PF_3_)(CH_2_) and Fe(CO)_3_(PF_3_)(N_2_); Table S1: Cartesian coordinates of all computed structures.
