# Supplementary material for: DFT Study on the Mechanism of Iron-Catalyzed Diazocarbonylation
Source: Molecules. 2020 Dec 11;25(24):5860. doi: 10.3390/molecules25245860 (PMC7763840; doi:10.3390/molecules25245860)
Supplement: Supplementary file 1 [file molecules-25-05860-s001.pdf]

Supplementary Material to Manuscript entitled "DFT Study on the Mechanism of Iron-Catalyzed Diazocarbonylation"

Tímea R. Kégl, László Kollár, Tamás Kégl

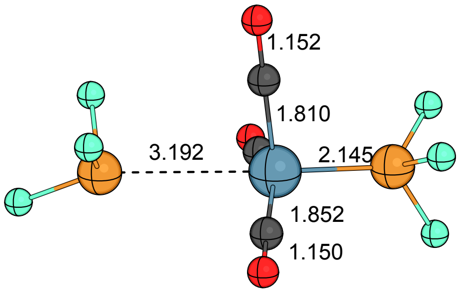

Figure S1: Computed structure of the triplet state adduct $Fe(CO)_3(PF_3)_2$. Distances are given in Å.

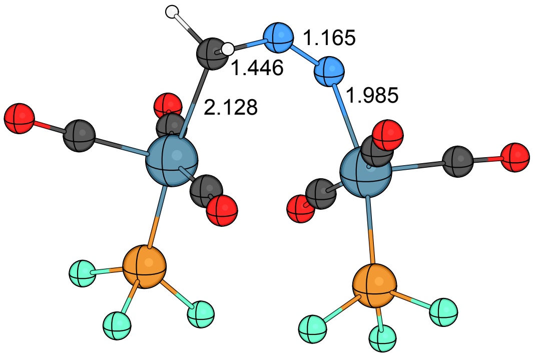

Figure S2: Computed structure of the triplet transition state for the associative pathway between complex **3F1** and $\cdot Fe(CO)_3(PF_3)$ leading to $Fe(CO)_3(PF_3)(CH_2)$ and $Fe(CO)_3(PF_3)(N_2)$. Distances are given in Å.

Table S1. Cartesian coordinates (in Å) of the computed structures occuring in this study

CO

C	0.00000	0.00000	-0.01697
O	0.00000	0.00000	1.11697

N_2

N	0.00000	0.00000	0.00060
N	0.00000	0.00000	1.09940

$N{=}N{=}CH_2$

C	0.00000	0.00000	1.24122
N	0.00000	0.00000	-0.05525
H	0.95457	-0.00000	1.74815
H	-0.95457	-0.00000	1.74815
N	-0.00000	-0.00000	-1.19563

$O{=}C{=}CH_2$

C	-0.00000	-0.00000	-1.21350
H	0.00000	0.94057	-1.75070
H	0.00000	-0.94057	-1.75070
C	-0.00000	-0.00000	0.10060
O	0.00000	0.00000	1.26812

PPh_3

P	-0.15309	0.14137	-1.24271
C	1.56806	-0.05142	-0.61403
C	1.94371	-0.96334	0.38128
C	2.55188	0.77518	-1.17998
C	3.27157	-1.04174	0.80446
H	1.19579	-1.61125	0.82824
C	3.87454	0.70713	-0.74647
H	2.27375	1.47817	-1.96280
C	4.23884	-0.20501	0.24663
H	3.54889	-1.75546	1.57663
H	4.62322	1.35888	-1.19007
H	5.27197	-0.26647	0.57900
C	-0.99420	-1.30037	-0.46273
C	-1.70029	-1.23906	0.74591
C	-0.91298	-2.52955	-1.13673
C	-2.30578	-2.38219	1.27061
H	-1.77442	-0.29635	1.27950
C	-1.50464	-3.67359	-0.60489
H	-0.37789	-2.58624	-2.08268
C	-2.20624	-3.60212	0.60089
H	-2.85204	-2.31891	2.20877
H	-1.42691	-4.61924	-1.13575
H	-2.67699	-4.49146	1.01205
C	-0.77004	1.53343	-0.20542
C	-0.08428	2.05355	0.90027
C	-2.00653	2.09633	-0.56020
C	-0.62619	3.10801	1.63718
H	0.87396	1.63131	1.18732
C	-2.55412	3.13895	0.18479
H	-2.54194	1.70875	-1.42476
C	-1.86285	3.65015	1.28566
H	-0.08181	3.50253	2.49190
H	-3.51573	3.55946	-0.09908
H	-2.28387	4.47006	1.86193

PF_3

P	0.00000	0.00000	0.50312
F	0.00000	1.38684	-0.27951
F	1.20104	-0.69342	-0.27951
F	-1.20104	-0.69342	-0.27951

1F

Fe	-0.00000	-0.00000	0.00000
C	-1.55487	-0.89761	0.00000
O	-2.55469	-1.47444	0.00000
C	0.00009	1.79535	-0.00000
O	0.00044	2.94966	-0.00000
C	1.55479	-0.89775	0.00000
O	2.55425	-1.47520	0.00000
P	-0.00000	-0.00000	2.11234
P	-0.00000	-0.00000	-2.11234
F	-1.19271	0.68861	2.86559
F	-0.00000	-1.37722	2.86559
F	1.19271	0.68861	2.86559
F	-0.00000	-1.37722	-2.86559
F	1.19271	0.68861	-2.86559
F	-1.19271	0.68861	-2.86559

2F

Fe	0.94714	0.00001	0.00001
C	0.98515	-1.25258	-1.30626
O	0.98195	-2.05203	-2.14014
C	0.98513	-0.50496	1.73791
O	0.98192	-0.82740	2.84719
C	0.98513	1.75756	-0.43163
O	0.98192	2.87944	-0.70704
P	-1.08624	-0.00000	-0.00000
F	-1.83818	0.38385	-1.32149
F	-1.83819	0.95250	0.99317
F	-1.83818	-1.33638	0.32829

2Ft

Fe	-0.81903	0.00013	-0.34809
C	-1.03938	1.78252	-0.79516
O	-1.15708	2.89982	-1.03927
C	-1.35985	0.00028	1.38195
O	-1.70403	0.00033	2.48209
C	-1.04004	-1.78217	-0.79515
O	-1.15828	-2.89943	-1.03922
P	1.27657	-0.00023	0.06906
F	2.31270	-0.00030	-1.12861
F	1.89552	-1.19430	0.89954
F	1.89589	1.19355	0.89971

3F1

Fe	-0.30741	0.26738	-0.00032
C	-0.58342	-0.53551	-1.57806
O	-0.79351	-1.05148	-2.59631
C	0.17019	1.99838	-0.00219
O	0.46626	3.11878	-0.00313
C	-0.58345	-0.53198	1.57923
O	-0.79352	-1.04543	2.59876
P	1.67054	-0.35003	0.00035
F	2.00230	-1.89126	0.00200
F	2.60880	0.07946	1.19168
F	2.60882	0.07691	-1.19188
C	-2.32640	0.96303	-0.00118
H	-2.52838	1.52651	-0.91376
H	-2.52843	1.52869	0.91005
N	-3.14206	-0.14443	0.00013
N	-3.72698	-1.09574	0.00119

3F2

Fe	0.50726	-0.35828	0.00268
O	0.64822	0.16651	2.88536
C	0.78391	1.12550	-1.53602
C	0.18849	-1.97919	-0.63851
O	0.00285	-3.07700	-0.96300
P	-1.56457	0.07352	0.00536
F	-2.52257	-0.63471	1.03026
F	-2.41326	-0.17029	-1.30842
F	-2.02367	1.57232	0.25665
H	-0.08741	1.28620	-2.17492
H	1.66822	0.85689	-2.11634
N	1.04570	2.29699	-0.87396
N	1.24725	3.22520	-0.27614
C	2.25998	-0.68071	-0.01316
O	3.39731	-0.88150	-0.03753
C	0.58374	0.00840	1.73879

3FN

Fe	-0.17843	0.52551	0.05791
C	0.41486	1.45854	-1.38984
O	0.79764	2.05992	-2.28980
C	0.32331	1.88775	1.08564
O	0.67723	2.75382	1.76247
C	-0.90326	-0.25522	1.50472
O	-1.39937	-0.74943	2.41798
P	1.31082	-0.95088	-0.11349
F	0.97840	-2.37604	-0.73081
F	2.00855	-1.47018	1.20904
F	2.61843	-0.65900	-0.95456
N	-1.80165	0.15225	-0.89858

N	-2.82755	-0.38998	-0.80123
C	-3.96810	-0.97200	-0.74749
H	-4.83048	-0.40675	-0.40853
H	-4.04140	-2.01116	-1.05252

3FNt

Fe	0.21491	0.00015	0.13540
C	0.56558	-1.78595	-0.17182
O	0.74986	-2.91291	-0.32744
C	-0.07194	0.00065	1.91675
O	-0.23709	0.00102	3.06014
C	0.56534	1.78609	-0.17288
O	0.74952	2.91298	-0.32916
P	-1.85508	-0.00025	-0.42640
F	-2.25605	-0.00103	-1.96205
F	-2.78615	1.19403	0.03853
F	-2.78611	-1.19407	0.03974
C	2.99536	0.00036	0.65150
H	3.04486	-0.93938	1.18727
H	3.04484	0.94067	1.18629
N	3.54598	-0.00027	-0.54129
N	3.90820	-0.00079	-1.61779

4TSF1

Fe	0.31462	0.32872	-0.00045
C	0.55094	-0.27891	1.66849
O	0.76277	-0.66408	2.74021
C	-0.17400	2.05091	-0.00286
O	-0.48003	3.16583	-0.00442
C	0.55094	-0.28359	-1.66769
O	0.76277	-0.67177	-2.73832
P	-1.63720	-0.45562	0.00063
F	-1.85939	-2.01901	0.00275
F	-2.60336	-0.10374	-1.19535
F	-2.60334	-0.10050	1.19567
C	2.18458	0.90773	-0.00126
H	2.59943	1.38223	0.89286
H	2.59942	1.37971	-0.89672
N	3.24059	-0.43471	0.00061
N	3.57867	-1.49127	0.00205

4TSF2

Fe	-0.66847	-0.06776	0.08498
O	-1.42671	-2.23102	-1.75429
C	-0.22819	1.45289	1.26019
C	-1.38561	-0.83135	1.53404
O	-1.87105	-1.32912	2.45736
P	1.36656	-0.60499	-0.09075
F	1.78988	-2.11165	-0.31062
F	2.36729	-0.25524	1.10113
F	2.26496	0.02375	-1.24812
H	0.49814	1.33152	2.06859
H	-1.03699	2.11251	1.58846
N	0.63593	2.67566	0.41918
N	1.09689	3.23563	-0.41869
C	-1.73398	1.02558	-0.85162
O	-2.41878	1.75336	-1.43484
C	-1.11488	-1.38779	-1.03194

5F1

Fe	0.71731	0.00015	-0.20349
C	0.88630	-1.78468	-0.35251
O	1.01442	-2.91982	-0.47107
C	1.22117	-0.00174	1.52823
O	1.61792	-0.00309	2.60917
C	0.88697	1.78499	-0.35002
O	1.01619	2.92009	-0.46769
P	-1.38386	0.00026	0.02900
F	-2.26704	-0.00234	-1.27807
F	-2.09424	1.19900	0.77071
F	-2.09437	-1.19546	0.77537
C	1.55949	0.00149	-1.84403
H	1.84834	-0.89263	-2.40537
H	1.84857	0.89625	-2.40423

5F2

Fe	-0.62264	-0.00072	0.17423
O	-0.83756	2.33786	-1.61311
C	-0.41484	-0.01034	2.00825
C	-0.74447	-1.39082	-0.96468
O	-0.83612	-2.32477	-1.63196
P	1.50636	-0.00013	0.13857
F	2.23612	0.00651	-1.25610
F	2.28311	-1.19321	0.80677
F	2.28339	1.18630	0.81817
H	0.52413	-0.01360	2.57561
H	-1.24290	-0.01365	2.72385
C	-2.36932	-0.00212	0.57250
O	-3.48493	-0.00318	0.84457
C	-0.74513	1.39867	-0.95335

6TSF1

Fe	0.73010	0.08045	-0.09903
C	1.36910	-1.55131	-0.67908
C	0.73622	1.85728	-0.14666
C	0.97631	-0.26065	1.64478
O	1.73561	-2.57449	-1.05126
O	1.16552	-0.43478	2.76757
O	0.60180	3.00236	0.07765
P	-1.31734	-0.21230	-0.06846
F	-2.15767	0.22879	-1.32725
F	-2.18134	0.50049	1.03964
F	-1.89833	-1.67007	0.09499
C	1.23188	1.12606	-1.60823
H	0.89890	1.94601	-2.24298
H	2.11028	0.64102	-2.03842

6TSF2

Fe	-0.59503	-0.06259	-0.07534
C	-0.66209	1.60192	-0.75059
C	-0.94456	0.41575	1.58316
O	-0.74592	2.65286	-1.21261
O	-1.20310	0.69079	2.67188
P	1.59261	-0.18512	-0.07824
F	2.31652	-0.18655	1.32422
F	2.47391	0.92044	-0.78198
F	2.29657	-1.46449	-0.69459
C	-0.95131	-1.91091	-0.31483
H	-0.12278	-2.34864	-0.87644
H	-1.64971	-2.68786	-0.00536
C	-2.17035	-0.72346	-0.53639
O	-3.30836	-0.87913	-0.77210

7F1

Fe	0.57607	0.59587	-0.00153
C	2.12059	-0.17418	-0.67265
C	-0.85697	1.80757	-0.26082
C	0.79446	0.33691	1.75274
O	3.00917	-0.74648	-1.12571
O	0.90398	0.22451	2.89539

O	-1.93176	2.19236	0.09511
P	-0.74047	-0.96244	-0.14406
F	-1.52878	-1.16800	-1.49158
F	-1.94760	-1.07668	0.85437
F	-0.17155	-2.42187	0.02481
C	0.02945	1.89843	-1.39874
H	-0.44990	2.03257	-2.36571
H	1.03409	2.33467	-1.36352

7F2

Fe	-0.52144	0.02128	-0.22699
C	-0.84009	1.74654	-0.60516
C	-1.03432	0.23473	1.41261
O	-1.10130	2.83883	-0.86415
O	-1.34527	0.37889	2.51625
P	1.65699	-0.11727	-0.06945
F	2.46689	-0.95644	-1.14681
F	2.18126	-0.84157	1.23253
F	2.60659	1.14624	0.00113
C	-0.94350	-1.91521	-0.47484
H	-1.04665	-2.74995	0.21758
H	-0.37382	-2.17273	-1.37260
C	-2.08514	-1.03847	-0.52497
O	-3.27232	-0.98912	-0.59321

9F1

Fe	-0.57077	0.15314	-0.02930
C	-0.54710	1.88703	-0.58864
C	-0.62009	-1.75896	-0.76274
C	-0.52654	0.06631	1.75702
O	-0.56932	2.97247	-0.96899
O	-0.53254	-0.00869	2.90708
O	-0.56399	-2.85773	-0.32010
P	1.56919	0.01709	-0.01178
F	2.35783	-0.18393	-1.36224
F	2.26771	-1.10492	0.83837
F	2.36419	1.25149	0.54974
C	-0.71959	-0.88454	-1.86257
H	0.13737	-0.79656	-2.52430
H	-1.68780	-0.79407	-2.34743
C	-2.36180	0.08092	0.06082
O	-3.50968	0.06200	0.12625

9F2

Fe	-0.45229	0.00384	0.06334
C	-0.61011	1.80308	0.03631
C	-2.18592	-0.03923	-1.04004
C	-0.84920	0.00948	1.80780
O	-0.70161	2.94881	0.02515
O	-1.12961	0.01399	2.92438
O	-3.34377	-0.06368	-0.80234
P	1.67894	0.01810	-0.13584
F	2.31840	-0.33156	-1.54196
F	2.54664	-0.97332	0.73937
F	2.49275	1.34389	0.14760
C	-1.10516	-0.02021	-1.94139
H	-0.88823	-0.93612	-2.48471
H	-0.93281	0.89825	-2.49636
C	-0.53903	-1.80035	0.06163
O	-0.58591	-2.94929	0.06791

1P

Fe	0.00077	0.00094	0.00129
C	0.00275	-0.03212	1.77533
C	-0.00085	1.55648	-0.85211
C	0.00036	-1.51739	-0.91752
O	0.00418	-0.05334	2.94118
O	0.00016	-2.51419	-1.52236
O	-0.00174	2.57913	-1.41224
P	-2.20596	-0.00177	0.00123
C	-2.99108	-1.09865	1.24559
C	-4.12904	-0.71146	1.96381
C	-2.44146	-2.36942	1.46622
C	-4.70866	-1.58256	2.88763
H	-4.56032	0.27180	1.80581
C	-3.02865	-3.24057	2.38052
H	-1.54746	-2.66744	0.92813
C	-4.16260	-2.84879	3.09571
H	-5.58681	-1.26776	3.44578
H	-2.59456	-4.22403	2.54165
C	-2.98830	-0.53231	-1.57168
C	-4.10658	-1.37426	-1.59950
C	-2.45272	-0.06471	-2.78029
C	-4.68082	-1.74199	-2.81754
H	-4.52679	-1.74722	-0.67107
C	-3.03497	-0.42394	-3.99322
H	-1.57376	0.57157	-2.76696
C	-4.14933	-1.26589	-4.01561
H	-5.54350	-2.40340	-2.82712
H	-2.61225	-0.05324	-4.92353
C	-2.99234	1.62368	0.32823
C	-2.46224	2.43541	1.34118
C	-4.10941	2.06744	-0.38970
C	-3.04868	3.66324	1.63744
H	-1.58445	2.10653	1.88793
C	-4.68800	3.30388	-0.09807
H	-4.52528	1.45032	-1.17961
C	-4.16190	4.10231	0.91689
H	-2.63024	4.28281	2.42650
H	-5.54984	3.64188	-0.66794
P	2.20758	-0.00033	-0.00244
C	2.99536	1.04390	1.28459
C	2.98995	0.59236	-1.55303
C	2.99009	-1.63970	0.25765
C	4.12262	0.62004	1.99875
C	2.45653	2.31190	1.54476
C	4.11775	1.42212	-1.54658
C	2.44607	0.18535	-2.77957
C	2.45695	-2.49132	1.23570
C	4.10616	-2.05660	-0.47766
C	4.70234	1.45224	2.95784
H	4.54552	-0.36144	1.81002
C	3.04396	3.14434	2.49415
H	1.57078	2.63822	1.00947
C	4.69298	1.83758	-2.74865
H	4.54442	1.74851	-0.60363
C	3.02912	0.59221	-3.97709
H	1.55990	-0.44088	-2.79175
C	3.03935	-3.73229	1.48069
H	1.57992	-2.18277	1.79537
C	4.68064	-3.30602	-0.23772
H	4.52434	-1.40833	-1.24096
C	4.16718	2.71593	3.20541
H	5.57209	1.10896	3.51233
H	2.61839	4.12605	2.68573
C	4.15287	1.42172	-3.96520
H	5.56307	2.48904	-2.73132
H	2.59966	0.26850	-4.92173
C	4.15146	-4.14452	0.74278
H	2.61858	-4.38295	2.24303
H	5.54161	-3.62270	-0.82099
H	-4.61189	5.06585	1.14193
H	-4.59596	-1.55448	-4.96364
H	-4.61335	-3.52563	3.81700
H	4.61798	3.36236	3.95403
H	4.60017	1.74763	-4.90074
H	4.59816	-5.11812	0.92740

2P

Fe	0.09820	0.01560	0.00748
C	0.06577	0.04041	1.80421
C	0.06948	1.56001	-0.91064
C	0.05029	-1.55210	-0.87001
O	0.06627	0.05796	2.96693
O	0.04066	-2.56659	-1.43829
O	0.07210	2.55947	-1.50505
P	2.24406	0.00316	0.00130
C	3.02206	1.05911	1.27900
C	3.00957	0.57648	-1.56005
C	3.00131	-1.64059	0.27851
C	4.11430	0.61160	2.03349
C	2.52589	2.35426	1.48779
C	4.09452	1.46260	-1.56274
C	2.50816	0.09891	-2.77989
C	2.50296	-2.45448	1.30640
C	4.07794	-2.09249	-0.49535
C	4.70143	1.44933	2.98258
H	4.50529	-0.38899	1.88208
C	3.12039	3.18940	2.42993
H	1.66795	2.70054	0.92134
C	4.66937	1.86380	-2.76942
H	4.48940	1.84053	-0.62564
C	3.09068	0.49515	-3.98074
H	1.65573	-0.57203	-2.78685
C	3.08028	-3.69566	1.56017
H	1.65692	-2.11943	1.89698
C	4.64774	-3.34110	-0.24276
H	4.47037	-1.47224	-1.29429
C	4.20797	2.73839	3.18150
H	5.54436	1.09028	3.56736
H	2.72769	4.19091	2.58406
C	4.17104	1.38064	-3.97887
H	5.50664	2.55685	-2.76087
H	2.69415	0.11923	-4.92012
C	4.15244	-4.14335	0.78470
H	2.68628	-4.31845	2.35885
H	5.47868	-3.68534	-0.85305
H	4.66424	3.38826	3.92365
H	4.61779	1.69690	-4.91786
H	4.59524	-5.11698	0.97777

2Pt

Fe	0.11023	-0.00563	-0.28706
C	-0.29897	0.00108	1.45457
C	-0.16435	1.74052	-0.75441
C	-0.09787	-1.77887	-0.70117
O	-0.50579	0.00429	2.59620
O	-0.21413	-2.90481	-0.93497
O	-0.33668	2.85073	-1.03296
P	2.33443	0.01691	-0.06342
C	3.06604	1.06361	1.25091
C	3.15674	0.58976	-1.59657
C	3.08732	-1.62043	0.25749
C	4.25359	0.69993	1.90214
C	2.44149	2.27035	1.59324
C	4.29916	1.39931	-1.59372
C	2.59597	0.19809	-2.82197
C	2.63700	-2.34125	1.37491
C	4.05833	-2.18609	-0.57654
C	4.80646	1.53270	2.87459
H	4.74312	-0.23568	1.65052
C	2.99955	3.10369	2.56105
H	1.51402	2.55380	1.10793
C	4.87366	1.80521	-2.79902
H	4.73562	1.71576	-0.65152
C	3.17703	0.59664	-4.02363
H	1.69877	-0.41791	-2.82669
C	3.15815	-3.60064	1.65588
H	1.87642	-1.91266	2.02208
C	4.57101	-3.45479	-0.29750
H	4.41404	-1.63791	-1.44320
C	4.18237	2.73656	3.20470
H	5.72519	1.23853	3.37564
H	2.50373	4.03591	2.81825
C	4.31744	1.40305	-4.01392
H	5.75747	2.43795	-2.78734
H	2.73515	0.28599	-4.96671
C	4.12529	-4.16268	0.81780
H	2.80358	-4.14881	2.52478
H	5.32154	-3.88718	-0.95431
H	4.61235	3.38275	3.96550
H	4.76624	1.72239	-4.95083
H	4.52489	-5.15018	1.03286

3P1

Fe	0.03982	0.03991	0.01059
C	0.03224	0.01277	1.79160
C	0.06439	1.56182	-0.91751
C	-0.00926	-1.50720	-0.87555
O	0.00888	0.00891	2.95851
O	-0.05636	-2.52010	-1.45089
O	0.06160	2.56074	-1.52037
P	2.22808	0.00355	0.00996
C	3.03478	1.05273	1.28224
C	3.01257	0.56558	-1.55062
C	2.99613	-1.64022	0.28654
C	4.19050	0.63998	1.95726
C	2.49144	2.31394	1.56281
C	4.06577	1.48749	-1.56545
C	2.53897	0.04656	-2.76462
C	2.44887	-2.48860	1.25895
C	4.12871	-2.05589	-0.42506
C	4.79350	1.47755	2.89664
H	4.61800	-0.33630	1.75310
C	3.10180	3.15210	2.49293
H	1.58283	2.62814	1.05934
C	4.63698	1.88394	-2.77618
H	4.43909	1.89941	-0.63375
C	3.11847	0.43544	-3.96932
H	1.71155	-0.65547	-2.76333
C	3.03163	-3.72619	1.52102
H	1.55947	-2.18172	1.79937
C	4.70462	-3.30063	-0.16679
H	4.55978	-1.40916	-1.18240
C	4.25344	2.73540	3.16401
H	5.68536	1.14315	3.42056
H	2.67161	4.12830	2.70121
C	4.16776	1.35784	-3.97891
H	5.44916	2.60647	-2.77555
H	2.74434	0.02454	-4.90346
C	4.15982	-4.13670	0.80749
H	2.59843	-4.37520	2.27768
H	5.57907	-3.61561	-0.73059
H	4.72269	3.38585	3.89767
H	4.61224	1.66836	-4.92097
H	4.60752	-5.10707	1.00615
C	-2.09572	-0.00996	-0.04406
H	-2.43987	0.03388	-1.07828
H	-2.45927	-0.87857	0.50669
N	-2.52713	1.11861	0.60019
N	-2.78798	2.06394	1.14060

3P2

Fe	-0.05563	-0.47360	-0.03450
C	-0.09479	-0.69106	1.70854
N	-0.14448	1.52289	-2.07019
C	0.08297	-1.83051	-1.15615
O	-0.14385	-0.96982	2.84149
O	0.14019	-2.79414	-1.80972

N	-0.14327	1.40509	-3.18900
P	2.18080	-0.34739	-0.01072
C	2.96929	0.70143	1.28179
C	2.81197	0.39587	-1.56455
C	3.09030	-1.92977	0.13127
C	4.35714	0.67508	1.48309
C	2.18675	1.56821	2.05231
C	3.27064	1.71910	-1.61419
C	2.69567	-0.32973	-2.76194
C	2.55694	-2.93160	0.95323
C	4.30976	-2.15521	-0.52190
C	4.94750	1.50525	2.43305
H	4.97697	-0.00093	0.90259
C	2.77920	2.40196	3.00205
H	1.10892	1.56753	1.92162
C	3.59803	2.30905	-2.83703
H	3.37932	2.28914	-0.69638
C	3.03887	0.25624	-3.97685
H	2.32748	-1.35048	-2.74252
C	3.24045	-4.13322	1.12828
H	1.60071	-2.77328	1.44108
C	4.98691	-3.36229	-0.35070
H	4.72415	-1.39459	-1.17654
C	4.15992	2.37354	3.19318
H	6.02331	1.47056	2.58477
H	2.15794	3.06477	3.59866
C	3.48385	1.58057	-4.02010
H	3.94857	3.33776	-2.86037
H	2.94502	-0.31804	-4.89463
C	4.45565	-4.35190	0.47722
H	2.81615	-4.90389	1.76619
H	5.92772	-3.52998	-0.86878
H	4.62165	3.01736	3.93726
H	3.73897	2.03949	-4.97142
H	4.98222	-5.29376	0.60740
C	-0.13867	1.58917	-0.70763
H	0.72776	2.17636	-0.40125
H	-1.08255	2.02275	-0.37483
C	-1.81973	-0.54303	-0.10697
O	-2.97833	-0.58172	-0.15415

3PN

Fe	-0.15486	-0.62805	-0.05912
C	-0.07927	-0.80537	1.70264
N	0.05607	2.08633	-1.28874
C	0.08519	-2.13274	-0.96604
O	-0.03771	-0.98765	2.84904
O	0.22443	-3.15714	-1.49452
N	-0.34698	1.08034	-0.86708
P	2.08777	-0.32701	-0.10756
C	2.76679	0.76036	1.20052
C	2.67910	0.44522	-1.66211
C	3.09669	-1.84493	0.04227
C	4.06700	0.58654	1.69369
C	1.97951	1.80927	1.69305
C	3.66397	1.44014	-1.66869
C	2.12268	0.02347	-2.87798
C	2.83108	-2.72236	1.10414
C	4.11782	-2.15238	-0.86420
C	4.57288	1.45531	2.66043
H	4.68020	-0.23049	1.32591
C	2.49025	2.67932	2.65430
H	0.96023	1.92514	1.33968
C	4.09282	1.99782	-2.87356
H	4.09069	1.78666	-0.73345
C	2.55351	0.58151	-4.07941
H	1.34112	-0.72963	-2.87849
C	3.58305	-3.88364	1.25808
H	2.03447	-2.49945	1.80643
C	4.86384	-3.32235	-0.71050
H	4.32903	-1.48204	-1.69089
C	3.78767	2.50486	3.13932
H	5.57981	1.30805	3.04237
H	1.86964	3.48691	3.03354
C	3.53987	1.57070	-4.08031
H	4.85485	2.77283	-2.86564
H	2.11405	0.24708	-5.01551
C	4.60001	-4.18802	0.34983
H	3.36874	-4.55675	2.08391
H	5.65042	-3.55533	-1.42355
H	4.18148	3.17835	3.89601
H	3.87066	2.00970	-5.01802
H	5.17970	-5.09988	0.46668
C	0.48198	3.20062	-1.77123
H	0.56327	4.06091	-1.11489
H	0.76486	3.24290	-2.81783
C	-1.92482	-0.90160	-0.05237
O	-3.05848	-1.11472	-0.03002

3PNt

Fe	0.03108	0.03634	0.02252
C	-0.07386	-0.47978	1.75043
C	0.00378	1.15818	-1.39915
C	-0.29800	-1.49988	-0.81361
O	-0.17747	-0.81167	2.85243
O	-0.50555	-2.50025	-1.36124
O	-0.04495	1.85059	-2.32030
P	2.27659	-0.08105	0.00420
C	2.99371	1.00367	1.29203
C	3.05183	0.48748	-1.55202
C	3.07531	-1.71007	0.26638
C	4.20363	0.71980	1.93840
C	2.28894	2.16955	1.62704
C	3.83521	1.64378	-1.62264
C	2.78911	-0.24042	-2.72312
C	2.42231	-2.70254	1.00762
C	4.35589	-1.97259	-0.24481
C	4.70419	1.59613	2.90104
H	4.74877	-0.18756	1.69842
C	2.79642	3.04579	2.58490
H	1.33351	2.38395	1.15211
C	4.35176	2.06602	-2.84971
H	4.04100	2.21501	-0.72292
C	3.31237	0.17915	-3.94213
H	2.17315	-1.13503	-2.67689
C	3.04161	-3.92870	1.24626
H	1.42429	-2.52466	1.39061
C	4.97188	-3.20017	-0.00762
H	4.86708	-1.21779	-0.83432
C	4.00492	2.76097	3.22282
H	5.64041	1.36574	3.40305
H	2.23877	3.94327	2.83844
C	4.09420	1.33602	-4.00888
H	4.95665	2.96791	-2.89558
H	3.10465	-0.39219	-4.84302
C	4.31717	-4.18028	0.74036
H	2.52115	-4.69034	1.82062
H	5.96277	-3.39174	-0.41129
H	4.39658	3.43980	3.97595
H	4.49598	1.66710	-4.96278
H	4.79613	-5.13906	0.92066
C	-1.97942	0.63638	0.28426
H	-2.52224	0.70890	-0.66390
H	-2.54840	-0.03922	0.93047
N	-2.09202	1.97433	0.90697
N	-1.30458	2.76139	1.24738

4TSP1

Fe	0.01052	0.04229	-0.00081
C	0.00860	-0.12346	1.77214
C	0.09171	1.50091	-1.02593
C	-0.09780	-1.48171	-0.91214

O	-0.03029	-0.19857	2.93438
O	-0.18559	-2.47921	-1.50496
O	0.09929	2.46210	-1.68262
P	2.23342	-0.01718	0.02207
C	3.03425	1.03859	1.29318
C	3.01853	0.53422	-1.54001
C	2.99839	-1.66309	0.29602
C	4.24308	0.68191	1.90443
C	2.42837	2.25545	1.63321
C	3.92446	1.59875	-1.58918
C	2.66258	-0.12070	-2.72902
C	2.37717	-2.57474	1.15935
C	4.21054	-2.01641	-0.31345
C	4.83627	1.53191	2.83823
H	4.71839	-0.26196	1.65774
C	3.02899	3.10820	2.55715
H	1.47892	2.52048	1.17757
C	4.46796	2.00335	-2.81042
H	4.20442	2.11553	-0.67711
C	3.21422	0.27859	-3.94275
H	1.95254	-0.94160	-2.70240
C	2.96338	-3.81242	1.41726
H	1.42705	-2.31995	1.61646
C	4.79151	-3.25872	-0.05916
H	4.69756	-1.32203	-0.99087
C	4.23379	2.74765	3.16341
H	5.76956	1.24069	3.31350
H	2.55003	4.05022	2.81132
C	4.11685	1.34467	-3.98741
H	5.16616	2.83605	-2.83762
H	2.93353	-0.23778	-4.85706
C	4.17092	-4.15819	0.80835
H	2.47017	-4.51134	2.08772
H	5.72831	-3.52360	-0.54287
H	4.69640	3.40756	3.89280
H	4.53971	1.66123	-4.93732
H	4.62212	-5.12771	1.00305
C	-1.93894	0.08439	-0.03347
H	-2.45908	0.26200	-0.98062
H	-2.50605	-0.63666	0.56470
N	-2.51634	1.46680	0.75589
N	-2.49321	2.42051	1.32557

4TSP2

Fe	-0.05714	-0.48834	-0.12079
C	-0.21508	-0.78746	1.60971
N	0.00129	1.77550	-2.13090
C	0.11377	-1.99637	-1.05488
O	-0.35642	-1.05093	2.73492
O	0.18693	-2.99801	-1.64009
N	-0.11678	1.53477	-3.21634
P	2.17646	-0.35066	-0.04685
C	2.94665	0.70222	1.25182
C	2.80383	0.36988	-1.60850
C	3.10338	-1.91990	0.13612
C	4.34339	0.75738	1.37772
C	2.15473	1.45265	2.12665
C	3.27393	1.68788	-1.68066
C	2.66082	-0.36852	-2.79536
C	2.61541	-2.87769	1.03561
C	4.29770	-2.17217	-0.55174
C	4.93237	1.55502	2.35518
H	4.97116	0.17015	0.71453
C	2.74603	2.25189	3.10666
H	1.07404	1.39759	2.04818
C	3.59518	2.25707	-2.91511
H	3.39335	2.27034	-0.77255
C	2.99319	0.19804	-4.02164
H	2.27806	-1.38380	-2.75800
C	3.31542	-4.06331	1.24787
H	1.68218	-2.69919	1.55977
C	4.99207	-3.36366	-0.34244
H	4.68036	-1.44470	-1.26080
C	4.13413	2.30617	3.22199
H	6.01517	1.58604	2.44534
H	2.11794	2.82527	3.78334
C	3.45613	1.51601	-4.08674
H	3.95424	3.28222	-2.95621
H	2.87856	-0.38531	-4.93128
C	4.50427	-4.31013	0.55893
H	2.92549	-4.79939	1.94579
H	5.91310	-3.55241	-0.88807
H	4.59472	2.92460	3.98798
H	3.70320	1.96012	-5.04718
H	5.04434	-5.23964	0.71883
C	-0.07468	1.46385	-0.50567
H	0.76187	2.10280	-0.20497
H	-0.99592	2.02781	-0.31733
C	-1.80172	-0.45917	-0.40475
O	-2.94260	-0.41167	-0.59892

5P1

Fe	-0.02005	-0.14108	-0.12115
C	-0.20014	-0.56624	1.60303
C	-0.13355	0.59829	-1.74812
C	-0.27514	-1.79919	-0.72833
O	-0.34229	-0.80158	2.72456
O	-0.52245	-2.85828	-1.12539
O	-0.24575	1.09680	-2.78105
P	2.20877	-0.13536	-0.04018
C	2.89482	0.93502	1.28639
C	3.01408	0.49327	-1.55934
C	3.06702	-1.73692	0.23063
C	4.13668	0.66636	1.87852
C	2.18070	2.07383	1.68076
C	3.74069	1.68825	-1.58445
C	2.84550	-0.23355	-2.74830
C	2.48237	-2.69663	1.06739
C	4.31602	-2.00965	-0.34647
C	4.65348	1.52584	2.84755
H	4.69719	-0.21670	1.58934
C	2.70503	2.93687	2.64183
H	1.20827	2.26758	1.23526
C	4.29231	2.14883	-2.78212
H	3.87746	2.25923	-0.67173
C	3.40460	0.22313	-3.93754
H	2.27393	-1.15822	-2.73880
C	3.13472	-3.89995	1.32862
H	1.51151	-2.50979	1.51225
C	4.96392	-3.21796	-0.09031
H	4.78105	-1.27821	-0.99951
C	3.94149	2.66407	3.22896
H	5.61320	1.30293	3.30675
H	2.14116	3.81726	2.93924
C	4.12870	1.41840	-3.95798
H	4.85223	3.08045	-2.79156
H	3.26883	-0.34908	-4.85143
C	4.37634	-4.16515	0.74882
H	2.66647	-4.63433	1.97853
H	5.92878	-3.41816	-0.54925
H	4.34497	3.33080	3.98668
H	4.55873	1.77861	-4.88877
H	4.88024	-5.10785	0.94520
C	-1.11805	1.26997	0.34265
H	-1.48265	2.04018	-0.34544
H	-1.52170	1.45807	1.34376

5P2

Fe	-0.08857	-0.62296	0.02807
C	-0.01505	-1.06062	1.75927
C	0.10236	-2.21899	-0.76483
O	-0.01321	-1.25580	2.90060

O	0.20769	-3.21525	-1.34281
P	2.14690	-0.29068	-0.04820
C	2.82044	0.75052	1.30169
C	2.77920	0.48947	-1.58188
C	3.14569	-1.82285	0.08399
C	4.13925	0.59174	1.75265
C	2.01538	1.74427	1.87200
C	3.80476	1.44157	-1.57210
C	2.23063	0.08375	-2.80765
C	3.03125	-2.59721	1.24925
C	3.97628	-2.26729	-0.95084
C	4.64349	1.41944	2.75451
H	4.76767	-0.18317	1.32427
C	2.52460	2.57356	2.87065
H	0.98759	1.85088	1.53795
C	4.28056	1.97406	-2.77163
H	4.22807	1.77429	-0.63005
C	2.71613	0.60789	-4.00337
H	1.41406	-0.63124	-2.81818
C	3.73693	-3.79027	1.37409
H	2.39520	-2.26523	2.06343
C	4.67663	-3.46884	-0.82518
H	4.07791	-1.67870	-1.85636
C	3.83794	2.41313	3.31402
H	5.66504	1.28488	3.10075
H	1.88985	3.33884	3.30955
C	3.74200	1.55570	-3.98806
H	5.07195	2.71891	-2.75193
H	2.28541	0.28408	-4.94717
C	4.55949	-4.23227	0.33470
H	3.64045	-4.37884	2.28263
H	5.31463	-3.80461	-1.63858
H	4.23066	3.05425	4.09896
H	4.11305	1.97212	-4.92091
H	5.10402	-5.16788	0.43062
C	-0.25091	1.05038	-0.71538
H	0.58362	1.68625	-1.03638
H	-1.19163	1.57273	-0.92652
C	-1.86207	-0.61601	-0.04007
O	-3.01368	-0.59320	-0.09550

6TSP1

Fe	0.02881	-0.39177	-0.19220
C	-0.21747	-0.87953	1.55022
C	-0.35791	0.57196	-1.62199
C	0.00750	-2.03107	-0.86576
O	-0.36476	-1.17859	2.65735
O	-0.04812	-3.08045	-1.35226
O	-0.46246	1.03412	-2.70114
P	2.20440	-0.24392	-0.18035
C	2.89325	0.76960	1.18610
C	2.91381	0.50497	-1.68936
C	3.12449	-1.82113	-0.00708
C	4.17484	0.51452	1.69614
C	2.15459	1.84342	1.69688
C	3.77347	1.60925	-1.64618
C	2.56306	-0.04458	-2.93214
C	2.77734	-2.68260	1.04470
C	4.16353	-2.18532	-0.87182
C	4.70591	1.32554	2.69811
H	4.75598	-0.31707	1.31003
C	2.69145	2.65762	2.69314
H	1.15442	2.02580	1.31285
C	4.27910	2.15095	-2.82902
H	4.04786	2.04690	-0.69199
C	3.07977	0.49046	-4.10839
H	1.87917	-0.88677	-2.97619
C	3.45764	-3.88316	1.22666
H	1.97502	-2.41322	1.72387
C	4.83779	-3.39475	-0.69226
H	4.44826	-1.52655	-1.68524
C	3.96687	2.39941	3.19747
H	5.69809	1.11629	3.08973
H	2.10819	3.48807	3.08249
C	3.93792	1.59144	-4.06004
H	4.94202	3.01127	-2.78497
H	2.79985	0.05675	-5.06455
C	4.48801	-4.24526	0.35528
H	3.17833	-4.54061	2.04571
H	5.63864	-3.66859	-1.37432
H	4.38083	3.02754	3.98201
H	4.33191	2.01555	-4.97988
H	5.01240	-5.18722	0.49259
C	-0.85919	1.30097	-0.14725
H	-0.91672	2.30144	-0.57237
H	-1.55043	1.21198	0.69317

6TSP2

Fe	-0.09995	-0.64670	0.03182
C	-0.41986	-0.89817	1.72832
C	-0.02228	-2.37853	-0.37472
O	-0.64288	-1.03393	2.85765
O	0.00004	-3.49428	-0.67987
P	2.17625	-0.37665	-0.05104
C	2.86954	0.67183	1.28554
C	2.74275	0.44101	-1.58966
C	3.20705	-1.88511	0.05383
C	4.18452	0.49907	1.74129
C	2.08083	1.68910	1.83773
C	3.59874	1.54823	-1.59822
C	2.26409	-0.06527	-2.80863
C	2.94424	-2.77777	1.10416
C	4.23132	-2.17797	-0.85366
C	4.70100	1.33622	2.72936
H	4.80165	-0.29165	1.32576
C	2.60247	2.52857	2.82133
H	1.05622	1.80946	1.49857
C	3.97685	2.13441	-2.80793
H	3.96784	1.95363	-0.66148
C	2.65280	0.51320	-4.01413
H	1.58211	-0.91240	-2.80698
C	3.69808	-3.93912	1.24600
H	2.14541	-2.56095	1.80844
C	4.97907	-3.34900	-0.71455
H	4.44579	-1.49348	-1.66829
C	3.91235	2.35360	3.26987
H	5.72015	1.19150	3.07867
H	1.98029	3.31257	3.24507
C	3.50968	1.61696	-4.01576
H	4.63786	2.99730	-2.80359
H	2.27975	0.10952	-4.95167
C	4.71639	-4.22940	0.33433
H	3.48524	-4.62317	2.06318
H	5.76897	-3.56999	-1.42789
H	4.31509	3.00207	4.04360
H	3.80502	2.07518	-4.95595
H	5.29868	-5.14088	0.44035
C	-0.49207	1.12474	-0.55173
H	0.31916	1.46549	-1.20176
H	-1.21684	1.92459	-0.39989
C	-1.67795	-0.13138	-0.55374
O	-2.81748	-0.03981	-0.83309

7P1

Fe	0.05795	-0.43906	-0.17729
C	-0.05612	-1.21374	1.48230
C	-0.30467	0.90534	-1.43336
C	0.14193	-1.87824	-1.16716
O	-0.11603	-1.71881	2.52184
O	0.16361	-2.81307	-1.85398
O	-0.08194	1.41623	-2.50361
P	2.22231	-0.22277	-0.18840

C	2.85694	0.82296	1.17675
C	2.94967	0.50953	-1.69380
C	3.12626	-1.80129	0.02484
C	4.13724	0.61653	1.71186
C	2.07605	1.88116	1.65687
C	3.94560	1.49384	-1.63083
C	2.50255	0.06493	-2.94676
C	3.04168	-2.47050	1.25610
C	3.84811	-2.38722	-1.02170
C	4.62395	1.45720	2.71174
H	4.75212	-0.19899	1.34449
C	2.56813	2.72377	2.65254
H	1.07951	2.03259	1.25148
C	4.50029	2.00613	-2.80290
H	4.28782	1.86186	-0.66951
C	3.06996	0.56909	-4.11417
H	1.70421	-0.66536	-3.00936
C	3.66761	-3.70107	1.43325
H	2.49535	-2.02585	2.08083
C	4.46761	-3.62485	-0.84245
H	3.93091	-1.87834	-1.97594
C	3.84116	2.51225	3.18392
H	5.61605	1.28694	3.12187
H	1.95296	3.54174	3.01827
C	4.06899	1.54051	-4.04567
H	5.27053	2.77071	-2.74259
H	2.71327	0.21740	-5.07829
C	4.37893	-4.28496	0.38222
H	3.59621	-4.20643	2.39277
H	5.02232	-4.06992	-1.66429
H	4.22078	3.16404	3.96644
H	4.50120	1.94287	-4.95820
H	4.86220	-5.24861	0.51982
C	-1.02341	1.25355	-0.25351
H	-1.44978	2.24599	-0.14663
H	-1.47458	0.54778	0.46589

7P2

Fe	0.04087	-1.03596	0.20622
C	0.16090	-1.02012	1.96458
C	-1.45064	-1.99794	0.24317
O	0.14894	-0.91298	3.12169
O	-2.39133	-2.66945	0.27713
P	2.20915	-0.37462	0.00521
C	2.88811	0.66074	1.34891
C	2.73883	0.50177	-1.51232
C	3.23450	-1.89518	0.00834
C	4.20028	0.50237	1.81218
C	2.07443	1.66052	1.90020
C	3.22456	1.81399	-1.48178
C	2.59290	-0.14587	-2.74979
C	2.83057	-2.98113	0.80069
C	4.42245	-1.99333	-0.73070
C	4.69287	1.33576	2.81562
H	4.83299	-0.27593	1.39647
C	2.57526	2.49711	2.89643
H	1.05082	1.77866	1.55821
C	3.56829	2.46450	-2.66861
H	3.33847	2.32761	-0.53270
C	2.94790	0.50117	-3.93004
H	2.20063	-1.15875	-2.78353
C	3.59945	-4.14284	0.84873
H	1.91561	-2.91098	1.38187
C	5.19030	-3.15586	-0.67666
H	4.74583	-1.16120	-1.34766
C	3.88311	2.33599	3.35645
H	5.70927	1.20122	3.17664
H	1.93719	3.26782	3.32025
C	3.43507	1.81035	-3.89242
H	3.94328	3.48393	-2.63212
H	2.83611	-0.01303	-4.88101
C	4.78041	-4.23310	0.11077

H	3.27352	-4.97760	1.46342
H	6.11083	-3.21891	-1.25127
H	4.26812	2.98293	4.14038
H	3.70496	2.31763	-4.81484
H	5.37912	-5.13936	0.14834
C	-0.55398	-0.17986	-1.52791
H	-1.45192	-0.55143	-2.02108
H	0.15957	0.27779	-2.21291
C	-0.78933	0.55008	-0.29656
O	-1.14605	1.57116	0.20910

8TSP

Fe	0.03021	-0.16726	-0.17042
C	-0.11433	-0.80919	1.53952
C	-0.22392	1.16941	-1.47358
C	0.02626	-1.65494	-1.10326
O	-0.19600	-1.22220	2.61796
O	-0.01018	-2.61165	-1.75873
O	0.02903	1.60354	-2.56952
P	2.19889	-0.09795	-0.18910
C	2.92495	0.87815	1.18176
C	2.96415	0.59058	-1.69435
C	2.98769	-1.74011	0.01126
C	4.21150	0.59030	1.66221
C	2.21482	1.95446	1.72574
C	3.96082	1.57421	-1.63787
C	2.52932	0.12453	-2.94454
C	2.82111	-2.42191	1.22721
C	3.70628	-2.35370	-1.02134
C	4.77494	1.37125	2.66977
H	4.77031	-0.24250	1.24676
C	2.78314	2.73668	2.73052
H	1.21300	2.16479	1.36286
C	4.53034	2.06320	-2.81295
H	4.29296	1.95860	-0.67943
C	3.11227	0.60458	-4.11408
H	1.72860	-0.60417	-3.00251
C	3.36025	-3.69314	1.40166
H	2.27887	-1.95601	2.04273
C	4.23923	-3.63157	-0.84493
H	3.85493	-1.83569	-1.96238
C	4.06249	2.44594	3.20564
H	5.77110	1.13861	3.03717
H	2.22211	3.56899	3.14738
C	4.11301	1.57490	-4.05167
H	5.30185	2.82688	-2.75802
H	2.76611	0.23615	-5.07578
C	4.06679	-4.30496	0.36330
H	3.22522	-4.20812	2.34913
H	4.79218	-4.09762	-1.65630
H	4.50167	3.05059	3.99485
H	4.55736	1.95872	-4.96634
H	4.48174	-5.30029	0.49828
C	-0.81272	1.65169	-0.26698
H	-0.95230	2.72021	-0.13020
H	-1.48095	1.06122	0.37775
C	-3.04783	-1.57056	0.22157
O	-3.13692	-2.70146	0.21399

9P1

Fe	-0.11350	-0.44139	-0.11137
C	-0.08915	-0.64323	1.68795
C	-0.29741	0.75541	-1.72001
C	0.03960	-2.01098	-0.93306
O	-0.14202	-0.80836	2.83230
O	0.11812	-3.00974	-1.51116
O	-0.44599	0.64400	-2.89722
P	2.15825	-0.26347	-0.11458
C	2.90386	0.73539	1.23653
C	2.83690	0.49094	-1.63479

C	3.06718	-1.84958	0.03829
C	4.27056	0.60575	1.52724
C	2.13561	1.65019	1.96570
C	3.48785	1.73057	-1.62685
C	2.62777	-0.16823	-2.85690
C	2.63260	-2.78238	0.99062
C	4.20762	-2.13319	-0.72411
C	4.85521	1.38225	2.52500
H	4.87632	-0.10801	0.97770
C	2.72357	2.42667	2.96466
H	1.07629	1.74613	1.75584
C	3.93280	2.29722	-2.82250
H	3.65152	2.25202	-0.68927
C	3.08526	0.39389	-4.04419
H	2.09761	-1.11537	-2.87879
C	3.32726	-3.97483	1.17663
H	1.74567	-2.58073	1.58198
C	4.89719	-3.33230	-0.54013
H	4.55668	-1.42126	-1.46462
C	4.08317	2.29608	3.24555
H	5.91375	1.26885	2.74403
H	2.11398	3.12921	3.52662
C	3.73724	1.62941	-4.03049
H	4.43323	3.26186	-2.80577
H	2.91641	-0.12510	-4.98358
C	4.46019	-4.25463	0.40993
H	2.97778	-4.69002	1.91638
H	5.77634	-3.54333	-1.14345
H	4.53938	2.89779	4.02725
H	4.08372	2.07219	-4.96049
H	4.99610	-5.18962	0.55013
C	-0.19617	1.60960	-0.59873
H	0.71954	2.18796	-0.50852
H	-1.09587	2.12823	-0.27621
C	-1.89112	-0.53381	-0.12299
O	-3.04256	-0.61284	-0.10850

9P2

Fe	-0.08528	-0.71215	-0.42440
C	-0.04370	-2.32640	0.36507
C	-1.79338	-0.36906	0.56603
C	-0.46780	-1.52355	-1.95329
O	-0.05623	-3.37406	0.84732
O	-0.77916	-2.05427	-2.93426
O	-2.95868	-0.52697	0.39144
P	2.16926	-0.36063	-0.28807
C	2.68062	0.83987	1.00834
C	2.97078	0.34545	-1.78407
C	3.19450	-1.83978	0.06116
C	3.68406	0.57530	1.94845
C	2.05498	2.09654	1.02354
C	4.08500	1.19098	-1.68538
C	2.49222	-0.00384	-3.05343
C	2.99838	-2.53285	1.26648
C	4.13430	-2.32660	-0.85600
C	4.04173	1.53963	2.89314
H	4.19745	-0.37988	1.94081
C	2.42150	3.06138	1.95786
H	1.29080	2.33051	0.29008
C	4.70149	1.68110	-2.83565
H	4.46809	1.46988	-0.70901
C	3.11559	0.48025	-4.20298
H	1.63283	-0.65911	-3.14319
C	3.73374	-3.67977	1.55176
H	2.27095	-2.17061	1.98708
C	4.86132	-3.48403	-0.57264
H	4.30045	-1.80350	-1.79179
C	3.41216	2.78313	2.90256
H	4.82072	1.31622	3.61766
H	1.93045	4.03099	1.95029
C	4.21957	1.32676	-4.09692
H	5.56010	2.34156	-2.74521
H	2.73187	0.20063	-5.18052
C	4.66633	-4.16144	0.63027
H	3.57152	-4.20287	2.49040
H	5.58417	-3.85270	-1.29585
H	3.69219	3.53306	3.63755
H	4.69976	1.71227	-4.99246
H	5.23412	-5.06178	0.84917
C	-0.74528	0.10111	1.39534
H	-0.62538	1.17575	1.50306
H	-0.50967	-0.47550	2.28632
C	-0.31149	0.87771	-1.22743
O	-0.45698	1.88683	-1.76987
